# TGIF2 is a potential biomarker for diagnosis and prognosis of glioma

**DOI:** 10.3389/fimmu.2024.1356833

**Published:** 2024-02-26

**Authors:** Wan Zhang, Long Zhang, Huanhuan Dong, Hang Peng

**Affiliations:** ^1^ Health Science Center of Xi’an Jiaotong University, Xi’an, Shaanxi, China; ^2^ Bone and Joints Research Center, The First Affiliated Hospital of Xi’an Jiaotong University, Xi’an, Shaanxi, China; ^3^ Department of Respiratory and Critical Care Medicine, The Second Affiliated Hospital of Xi’an Jiaotong University, Xi’an, Shaanxi, China; ^4^ Department of Thoracic Surgery, The First Affiliated Hospital of Xi’an Jiaotong University, Xi’an, Shaanxi, China; ^5^ Second Department of General Surgery, Shaanxi Provincial People’s Hospital, Xi’an, Shaanxi, China

**Keywords:** TGIF2, biomarker, glioma, immune infiltration, EMT

## Abstract

**Background:**

TGFB-induced factor homeobox 2 (TGIF2), a member of the Three-Amino-acid-Loop-Extension (TALE) superfamily, has been implicated in various malignant tumors. However, its prognostic significance in glioma, impact on tumor immune infiltration, and underlying mechanisms in glioma development remain elusive.

**Methods:**

The expression of TGIF2 in various human normal tissues, normal brain tissues, and gliomas was investigated using HPA, TCGA, GTEx, and GEO databases. The study employed several approaches, including Kaplan-Meier analysis, ROC analysis, logistic regression, Cox regression, GO analysis, KEGG analysis, and GSEA, to explore the relationship between TGIF2 expression and clinicopathologic features, prognostic value, and potential biological functions in glioma patients. The impact of TGIF2 on tumor immune infiltration was assessed through Estimate, ssGSEA, and Spearman analysis. Genes coexpressed with TGIF2 were identified, and the protein-protein interaction (PPI) network of these coexpressed genes were constructed using the STRING database and Cytoscape software. Hub genes were identified using CytoHubba plugin, and their clinical predictive value was explored. Furthermore, *in vitro* experiments were performed by knocking down and knocking out TGIF2 using siRNA and CRISPR/Cas9 gene editing, and the role of TGIF2 in glioma cell invasion and migration was analyzed using transwell assay, scratch wound-healing assay, RT-qPCR, and Western blot.

**Results:**

TGIF2 mRNA was found to be upregulated in 21 cancers, including glioma. High expression of TGIF2 was associated with malignant phenotypes and poor prognosis in glioma patients, indicating its potential as an independent prognostic factor. Furthermore, elevated TGIF2 expression positively correlated with cell cycle regulation, DNA synthesis and repair, extracellular matrix (ECM) components, immune response, and several signaling pathways that promote tumor progression. TGIF2 showed correlations with Th2 cells, macrophages, and various immunoregulatory genes. The hub genes coexpressed with TGIF2 demonstrated significant predictive value. Additionally, *in vitro* experiments revealed that knockdown and knockout of TGIF2 inhibited glioma cell invasion, migration and suppressed the epithelial-mesenchymal transition (EMT) phenotype.

**Conclusion:**

TGIF2 emerges as a potential biomarker for glioma, possibly linked to tumor immune infiltration and EMT.

## 1 Introduction

Glioma is the most prevalent primary brain tumor, accounting for approximately 80% of malignant primary brain tumors in adults (1). The World Health Organization (WHO) classifies gliomas into Grade II to IV based on malignancy, with Grade II representing low-grade gliomas and Grades III and IV representing high-grade gliomas. Low-grade gliomas have the potential to progress to high-grade gliomas (2, 3). Gliomas exhibit high invasiveness, heterogeneity, and poor marginal restriction, rendering them susceptible to recurrence. Consequently, controlling invasive lesions poses a significant challenge in glioma treatment. Despite the implementation of diverse treatment modalities, such as surgery, radiotherapy, chemotherapy, and immunotherapy, the overall prognosis for glioma patients remains bleak, with a median survival of only 15 months for the most malignant glioblastoma multiforme (Grade IV glioma) (4). Given these challenges, the identification of more sensitive molecular targets as biomarkers for glioma diagnosis, treatment, and prognosis assessment is imperative.

TGF-β-induced factor homeobox 2 (TGIF2) belongs to the three-amino-acid-loop extension (TALE) superfamily of homeodomain proteins (5). Operating as a transcriptional repressor, TGIF2 regulates the BMP/TGF-β pathway or directly binds to DNA (6–8). TGIF2 has been implicated in the development and progression of various cancers. For instance, in cervical cancer, TGIF2 downregulates FCMR by binding directly to its promoter, thereby promoting cervical cancer metastasis (9). In lung cancer, TGIF2 has been found to mediate the EGFR-RAS-ERK signaling pathway, enhancing the stemness of lung adenocarcinoma cells and facilitating LUAD progression and metastasis (10). MicroRNA-148a was found to inhibit ovarian cancer cell proliferation and invasion by suppressing TGIF2 (11). Additionally, TGIF2 is associated with the epithelial-mesenchymal transition (EMT) phenotype in various diseases, including lung adenocarcinoma (12), primary biliary cholangitis (13), chronic obstructive pulmonary disease (14), colon cancer (15), prostate cancer (16), hepatocellular carcinoma (17) *etc.* Notably, as a downstream target of multiple non-coding RNAs, TGIF2 regulates the progression of glioma (18–21). Previous *in vitro* experiments have suggested that TGIF2 promotes the EMT phenotype and migration in U87MG and A172 glioma cells (21), indicating the potential involvement of TGIF2 in regulating glioma cell invasion and tumor metastasis. However, whether TGIF2 can serve as a biomarker for tumor progression, diagnosis, and prognosis in glioma remains unclear.

In this study, we systematically analyzed transcriptomic data and clinicopathologic information of gliomas sourced from online databases. Our results revealed a significant overexpression of TGIF2 in gliomas compared to normal human brain tissues. Moreover, elevated TGIF2 expression correlated with malignant phenotypes and poor prognosis in glioma patients, establishing it as a potential independent prognostic indicator. Differentially expressed genes enrichment analysis uncovered positive associations between high TGIF2 expression and crucial biological processes, including cell cycle regulation, DNA synthesis and repair, extracellular matrix, immune response, and various signaling pathways implicated in tumor progression. Immune infiltration analysis revealed the association of TGIF2 with the infiltration status of multiple immune cells, including Th2 cells and macrophages. Furthermore, a comprehensive analysis of the TGIF2 coexpression gene network in glioma was conducted, shedding light on the expression patterns of genes interacting with TGIF2 and the potential clinical relevance of hub genes. Finally, knockdown and knockout of TGIF2 inhibited invasion, migration and EMT of U251 cells *in vitro*, suggesting its potential function in glioma invasion and migration. These findings collectively position TGIF2 as a promising candidate for a diagnostic and prognostic biomarker in glioma offering valuable insights into its multifaceted role in the regulation of glioma progression.

## 2 Materials and methods

### 2.1 Datasets collection

The RNA-seq data for the 33 cancers and normal tissue in this study were obtained from The Cancer Genome Atlas (TCGA) database (https://portal.gdc.cancer.gov/) and Genotype-Tissue Expression (GTEx) database (https://gtexportal.org/), in which contained 1152 normal human brain tissue and 706 glioma samples. Clinicopathologic information of glioma patients were acquired from TCGA database (comprising 699 tumor samples). The raw data were normalized with the transcripts per million (TPM) and subsequent analyses were performed using log2 (TPM+1). Expression profiling microarray data of GSE14805 were downloaded from the Gene Expression Omnibus (GEO) database (http://www.ncbi.nlm.nih.gov/projects/geo/) (containing 34 tumor samples and 4 normal samples) and statistically analyzed by Xiantao database (https://www.xiantaozi.com/), an online bioinformatic analysis tool based on R software.

### 2.2 TGIF2 expression analysis

The mRNA expression data of TGIF2 in normal tissues and subcellular localization of TGIF2 protein in SH-SY5Y cell line were sourced from Human Protein Atlas (HPA) database (http://www.proteinatlas.org/). Statistical analysis of TGIF2 expression in normal, tumor, and various clinicopathologic characteristic groups was performed and visualized by Xiantao tool (“stats”, “car” and “ggplot2” packages).

### 2.3 Survival analysis

Glioma patients were categorized into two groups based on the median expression of TGIF2 mRNA (low-expression group: 0%-50%; high-expression group: 50%-100%). Kaplan-Meier analysis and Cox regression analysis were employed for survival analysis. Overall survival (OS), disease-specific survival (DSS), and progression-free interval (PFI) were compared between the high and low TGIF2 expression groups by Xiantao tool (“Survival” package). Additionally, OS, DSS and PFI were compared in different clinicopathologic characteristic subgroups of glioma patients. Kaplan-Meier plots were generated by Xiantao tool (“Survminer” and “ggplot2” packages).

### 2.4 Receiver operator characteristic curve

Receiver Operator Characteristic (ROC) curves were utilized to assess the diagnostic value of TGIF2 and previously reported prognostic genes (IDH1, IDH2, EGFR, TP53, CDC6, CDC14B, and CHD5) in glioma. ROC analysis and visualized with Xiantao tool (“pROC”, “timeROC” and “ggplot2” packages). The closer the area under the ROC curve (AUC) is to “1”, the higher the diagnostic value of the gene.

### 2.5 Univariate logistic regression analysis

Univariate logistic regression analysis was performed using Xiantao tool (“stats” package). Glioma patients were divided into two groups according to the median mRNA expression of TGIF2, with the low-expression group serving as the control. Logistic regression models were constructed to assess the predictive effects of TGIF2 expression on different clinicopathologic characteristics. P value < 0.05 was considered statistically significant.

### 2.6 Univariate and multivariate cox regression analysis

Proportional hazards hypothesis testing was conducted using Xiantao tool (“survival” package), followed by univariate and multivariate Cox regression analyses to ascertain the independent prognostic significance of high TGIF2 expression and different clinicopathologic characteristics in the OS of glioma patients. Clinicopathologic factors with p value < 0.1 in the univariate Cox regression analysis were incorporated into the multivariate Cox regression analysis to further delineate their autonomous prognostic value. P value < 0.05 was considered as of statistical significance. Forest plots, risk score curve and point of survival chart were drawn using Xiantao tool (“ggplot2” package).

### 2.7 Prognostic model generation and prediction

Utilizing the independent prognostic factors identified post multivariate Cox regression analysis, Xiantao tool (“rms” package) was employed to construct nomogram model predicting 1-, 3-, and 5-year OS in glioma patients. Calibration analysis and visualization were carried out using Xiantao tool (“rms” package), depicting the differences between the predicted and actual probabilities corresponding to the model at different time points.

### 2.8 Differentially expressed genes analysis

For TCGA RNA-seq data, glioma patients were categorized into two groups (low-expression group: 0%-50%; high-expression group: 50%-100%) based on the median expression of TGIF2 mRNA. Differentially Expressed Genes (DEGs) of TCGA RNA-seq data and GSE14805 dataset statistically analyzed using Xiantao tool (“DEseq2” and “limma” packages respectively). Genes with an absolute log2 fold change (FC) > 1 and adjusted p value < 0.05 were considered DEGs and used for subsequent analysis. Volcano plots were generated by Xiantao tool (“ggplot2” package).

### 2.9 Gene ontology, Kyoto Encyclopedia of Genes and Genomes and gene set enrichment analysis

To determine the differences in biological functions and signaling pathways between high and low TGIF2 expression groups, the identified DEGs were analyzed for Gene Ontology (GO) and Kyoto Encyclopedia of Genes and Genomes (KEGG) enrichment using Xiantao tool (“clusterProfiler” package). Items with adjusted p value < 0.05 were considered as significant enrichment. The gene set “c2.cp.all.v2022.1.Hs.symbols.gmt [All Canonical Pathways]” in Molecular Signatures Database (MSigDB) was chosen as the reference gene set for Gene Set Enrichment Analysis (GSEA), and the items with false discovery rate (FDR) < 0.25 and adjusted p value < 0.05 were considered significantly enriched. Results of GO, KEGG and GSEA enrichment analyses were used Xiantao tool (“ggplot2” package) for visualization.

### 2.10 Immune infiltration analysis

The “Estimate” package in R software (v 4.2.1) was applied to assess the stromal score, immune score, and estimate score in the glioma microenvironment grouped by high and low TGIF2 expression. Single sample Gene Set Enrichment Analysis (ssGSEA) was used to analyze the immune infiltration of 24 immune cells in glioma by calculating the enrichment score of each immune cell in the tumor samples according to the marker genes of the immune cells by Xiantao tool (“GSVA” package). Wilcoxon rank-sum test compared immune cell enrichment scores between high and low TGIF2 expression groups. Correlation between TGIF2 and various immune cells and immunoregulatory genes was implemented by Spearman analysis (22, 23). Visualization was performed using Xiantao tool (“ggplot2” package).

### 2.11 Screening of TGIF2-coexpressed genes in glioma

Spearman’s correlation test was employed to analyze the correlation of each gene with TGIF2. Genes with adjusted p value < 0.05 were sorted by the correlation coefficient, and the top 50 genes positively or negatively correlated with TGIF2 were selected for subsequent analysis. The coexpression heatmap of the top 15 genes positively or negatively correlated with TGIF2 were visualized using Xiantao tool (“ggplot2” package).

### 2.12 Protein-protein interaction network construction and hub genes identification

To investigate the interaction relationships among coexpression genes correlated with TGIF2, the STRING database (https://string-db.org/) was used to establish a Protein-Protein Interaction (PPI) network based on default parameters. Subsequently, the network was visualized by the Cytoscape software, and the top 10 hub genes were identified using the CytoHubba plugin. Kaplan-Meier plots and ROC plots of these 10 hub genes were generated as previously described.

### 2.13 Cell culture, vector construction and transfection

U251 and 293T cells were cultured in DMEM (Cellmax, CGM102.05) medium supplemented with 10% FBS (Gibco, 2437694). To knockdown TGIF2, U251 cells were transfected with siRNA of TGIF2 (Sigma, SASI_Hs01_00107440) using Lipofectamine 3000 (Invitrogen, L3000015) at a final concentration of 100 pmol according to the manufacturer’s instructions when the cells reached approximately 50% confluence. For TGIF2 knockout, guide RNAs (gRNAs) targeting exon 2 of TGIF2 were designed using the http://crispor.tefor.net/website. The double-stranded DNA formed after annealing gRNA33 and gRNA140 were inserted into the PX459 V2.0 plasmid. We transfected 293T cells with the cloned vectors, extracted genomic DNA and performed Sanger sequencing to determine the knockout efficiency of the vectors. The screened gRNA was transfected into U251 cells, and the monoclones that successfully knocked out TGIF2 were selected for expansion culture for subsequent experiments. The primer sequences for synthesizing gRNA were as follows: gRNA33, Forward: 5′- CACCGACCTAGATCACTGTCCGACA -3′;Reverse: 5′-AAACTGTCGGACAGTGATCTAGGTC -3′. gRNA140, Forward: 5′- CACCGCGGTGAAGATCCTCCGGGAC -3′;Reverse: 5′- AAACGTCCCGGAGGATCTTCACCGC -3′. The primer sequences for verifying knockout efficiency of gRNA were as follows: Forward: 5′- CATCCCCTGTGTCCCTTGTC -3′;Reverse: 5′- ACTTGCAGCACTGACAGGTT -3′.

### 2.14 RNA extraction, cDNA synthesis, and RT-qPCR

Total RNA was extracted with TsingZol total RNA extraction reagent (TSINGKE, TSP401) and cDNA was synthesized using 5× All-In-One RT Master Mix (Applied Biological Materials, G490) following the manufacturer’s protocol. RT-qPCR was performed using PowerUp™ SYBR™ Green Master Mix (Applied Biosystems™, A25742) and PIKOREAL 96 Real-Time PCR System (ThermoFisher). GAPDH served as internal control for quantifying relative gene expression levels using the 2 –ΔΔCT method. The primer sequences were as follows: TGIF2, Forward: 5′- CTTCAACACGCCACCACCCACACC-3′; Reverse: 5′- CCTCTGTAGCGCCACCTCCACCAG -3′.CDH2, Forward: 5′- CCTCCAGAGTTTACTGCCATGAC -3′; Reverse: 5′- GTAGGATCTCCGCCACTGATTC -3′. TWIST1, Forward: 5′- GCCAGGTACATCGACTTCCTCT -3′; Reverse: 5′- TCCATCCTCCAGACCGAGAAGG -3′. TWIST2, Forward: 5′- GCAAGATCCAGACGCTCAAGCT -3′; Reverse: 5′- ACACGGAGAAGGCGTAGCTGAG -3′. GAPDH, Forward: 5′- GGAGTCCACTGGCGTCTTCA -3′; Reverse: 5′- GTCATGAGTCCTTCCACGATACC -3′.

### 2.15 Transwell assay and scratch wound-healing assay

For transwell assay, 5x10^4^ cells were inoculated in transwell (Millicell, PI8P01250) upper chamber containing serum-free DMEM, and the lower chamber was layered with DMEM containing 10% FBS. After 24 h of incubation at 37°C, non-invasive cells on the upper surface of the membrane were wiped off with a cotton swab. Subsequently, invasive cells into the lower surface of the membrane were fixed with 4% paraformaldehyde and stained with 0.1% crystalline violet (Solarbio, G1063), observed under the microscope and selected random fields were photographed. For scratch wound-healing assay, cells were plated into 12-well plates and cultured until 90% confluence, scratched using a 10 μL pipette tip, and washed with PBS to remove dropped cells. The cells migration distance was observed at 0 h and 24 h of scratching and photographed.

### 2.16 Western blot analysis

Cells were lysed with RIPA lysis buffer (Beyotime, P0013B) supplemented with 1 mM of the protease inhibitor phenylmethylsulfonyl fluoride (Beyotime, ST506) for 20 min on ice. After centrifugation at 12,000 rpm, the supernatant was collected, loaded onto a 10% polyacrylamide SDS gel, and subsequently transferred to a PVDF membrane (Millipore, IPVH00010). Membranes were blocked with TBST containing 5% skimmed milk (BD Pharmingen™, 232100) for 1 h at room temperature, followed by overnight incubation at 4°C with primary antibodies. After 5 washes with TBST, the membranes were incubated with secondary antibody at room temperature for 2 h. The membranes were then scanned and analyzed using the ChemiDoc™ system (Bio-Rad). The following antibodies were used: TGIF2 (11522-1-AP, Proteintech); GAPDH (EPR16891, Abcam); N-Cadherin (22018-1-AP, Proteintech); secondary antibody (AB0101, Abways).

### 2.17 Statistical analysis

Bioinformatics analysis was performed with Wilcoxon rank sum test to detect statistical significance between the two groups. The relationship between TGIF2 expression and baseline clinicopathologic characteristics of patients was evaluated using the chi-square test by Xiantao tool (“stats” package). Spearman’s correlation coefficient assessed the correlation of TGIF2 expression with various immune cells and other genes. Statistical analysis of *in vitro* experiments were performed with GraphPad Prism 7.0 and comparisons between two conditions were applied by Student’s t-test and the data were presented as mean ± standard deviation (SD). P value < 0.05 was considered statistically significant.

## 3 Results

### 3.1 TGIF2 expression is elevated in glioma and associated with worse prognosis

**Figure 1:**
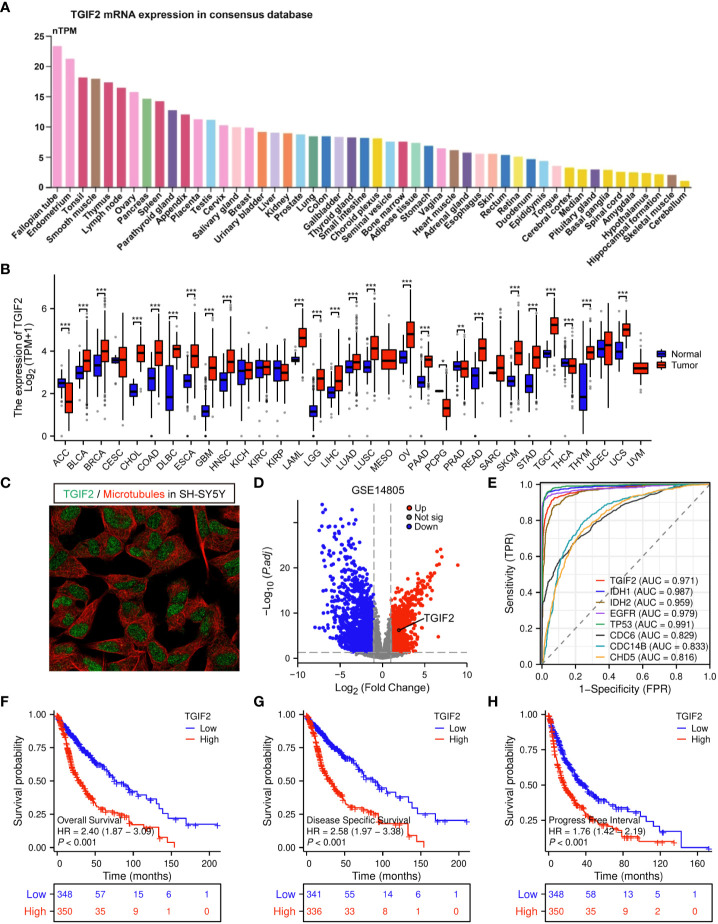
The expression level of TGIF2 in different human cancers and its relationship to glioma prognosis. **(A)** TGIF2 mRNA expression across various human organs and tissues based on consensus dataset in HPA database. **(B)** Comparison of TGIF2 expression between normal and tumor tissues across 33 cancers in TCGA and the GTEx database. **(C)** Subcellular localization of TGIF2 in SH-SY5Y cells from HPA datasets. **(D)** The volcano plot of DEGs in GSE14805. Red points represent upregulated, blue points represents downregulated genes. **(E)** ROC curve of TGIF2 and other established prognostic genes (IDH1, IDH2, EGFR, TP53, CDC6, CDC14B, CHD5) in glioma. **(F–H)** Survival curves for OS **(F)**, DSS **(G)** and PFI **(H)** in gliomas using TCGA database. ∗p < 0.05, ∗∗p < 0.01, ∗∗∗p < 0.001.

We explored the basal expression levels of TGIF2 across various tissues. Analysis of HPA database revealed higher TGIF2 mRNA expression in breast and female reproductive system, bone marrow, lymphoid, and muscle tissues, while lower expression was observed in brain tissues (**Figure 1A**). Subsequently, TGIF2 expression was assessed in 33 tumors from TCGA and GTEx databases. Notably, TGIF2 expression levels in 21 tumor tissues were significantly higher than in corresponding normal tissues (**Figure 1B**). Specifically, elevated TGIF2 expression was observed in low-grade glioma (LGG) and glioblastoma (GBM) compared to normal brain tissues (**Figure 1B**). Subcellular localization of TGIF2 protein is predominantly in the nucleus in the human glioma cell line SH-SY5Y (**Figure 1C**). Furthermore, in the GSE14805 dataset, we identified 2954 DEGs, including 1369 upregulated and 1585 downregulated genes, with TGIF2 significantly upregulated in the tumor group (**Figure 1D**). The ROC analysis indicated similar diagnostic ability for TGIF2 (AUC = 0.971) with established glioma prognostic genes (IDH1, IDH2, EGFR, TP53, CDC6, CDC14B, CHD5) (24–30) (**Figure 1E**). Kaplan-Meyer analysis demonstrated that glioma patients with high TGIF2 expression exhibited poor OS, DSS and PFI (**Figures 1F–H**). These findings suggest that TGIF2 expression is elevated in glioma and associated with worse prognosis, warranting further investigation.

### 3.2 High TGIF2 expression associated with malignant phenotypes of glioma

**Figure 2:**
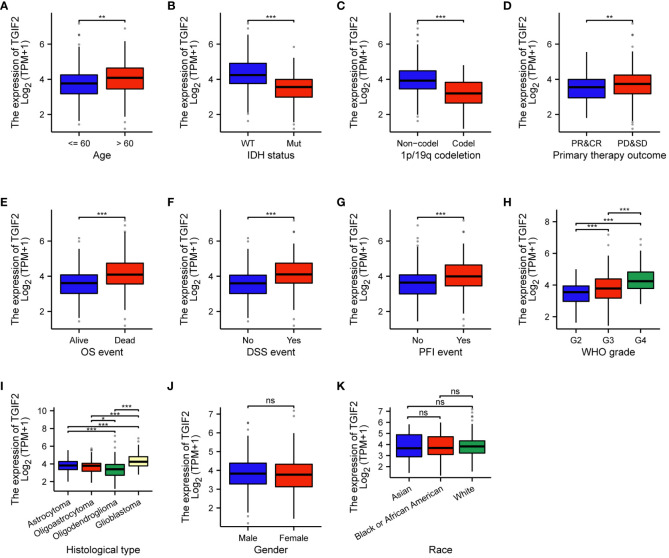
Associations between TGIF2 expression and various clinicopathologic characteristics in glioma. **(A)** Age. **(B)** IDH status. **(C)** 1p/19q codeletion status. **(D)** Primary therapy outcome. **(E)** OS events. **(F)** DSS events. **(G)** PFI events. **(H)** WHO grade. **(I)** Histological type. **(J)** Gender. **(K)** Race. ∗p < 0.05, ∗∗p < 0.01, ∗∗∗p < 0.001, ns, not significant.

We investigated the association between TGIF2 expression and various clinicopathologic characteristics of glioma patients in the TCGA database. A total of 699 patients’ clinicopathologic information was included in the statistics, and the chi-square test revealed that TGIF2 expression was significantly correlated with age, WHO grade, IDH status, 1p/19q codeletion, histological type, OS event, DSS event and PFI event in glioma patients (**Table 1**). Logistic regression analysis further confirmed significant associations between TGIF2 and age, WHO grade, IDH status, 1p/19q codeletion and histological type (**Table 2**). TGIF2 expression patterns in different clinicopathologic characteristics showed elevation in age > 60 years, IDH-wild type, 1p/19q non-codeletion, progressive disease and stable disease (PD&SD), OS events, DSS events, and PFI events subgroups (**Figures 2A–G**). TGIF2 expression increased with WHO grade classification, peaking in G4 glioma (**Figure 2H**). For histological type, glioblastoma exhibited significantly higher TGIF2 expression compared to other glioma types (**Figure 2I**). However, no statistically significant differences were observed among glioma patients of different genders and races (**Figures 2J, K**). These results suggest that high TGIF2 expression may be associated with malignant phenotypes of glioma and worsening clinical outcomes.

**Table 1:** Clinical characteristics of glioma patients.

Characteristics	Low TGIF2	High TGIF2	P value
n	349	350	
**Age, n (%)**			**0.007**
<= 60	292 (41.8%)	264 (37.8%)	
> 60	57 (8.2%)	86 (12.3%)	
**Gender, n (%)**			0.519
Male	196 (28%)	205 (29.3%)	
Female	153 (21.9%)	145 (20.7%)	
**WHO grade, n (%)**			**< 0.001**
G2	143 (22.4%)	81 (12.7%)	
G3	128 (20.1%)	117 (18.4%)	
G4	45 (7.1%)	123 (19.3%)	
**IDH status, n (%)**			**< 0.001**
WT	69 (10%)	177 (25.7%)	
Mut	274 (39.8%)	169 (24.5%)	
**1p/19q codeletion, n (%)**			**< 0.001**
Non-codel	221 (31.9%)	299 (43.2%)	
Codel	127 (18.4%)	45 (6.5%)	
**Histological type, n (%)**			**< 0.001**
Astrocytoma	95 (13.6%)	101 (14.4%)	
Oligoastrocytoma	72 (10.3%)	63 (9%)	
Oligodendroglioma	137 (19.6%)	63 (9%)	
Glioblastoma	45 (6.4%)	123 (17.6%)	
**Race, n (%)**			0.629
Asian	7 (1%)	6 (0.9%)	
Black or African American	19 (2.8%)	14 (2%)	
White	316 (46.1%)	324 (47.2%)	
**Primary therapy outcome, n (%)**			0.065
PD	53 (11.4%)	59 (12.7%)	
SD	89 (19.1%)	59 (12.7%)	
PR	40 (8.6%)	25 (5.4%)	
CR	88 (18.9%)	52 (11.2%)	
**OS event, n (%)**			**< 0.001**
Alive	254 (36.3%)	173 (24.7%)	
Dead	95 (13.6%)	177 (25.3%)	
**DSS event, n (%)**			**< 0.001**
Yes	82 (12.1%)	162 (23.9%)	
No	260 (38.3%)	174 (25.7%)	
**PFI event, n (%)**			**< 0.001**
Yes	144 (20.6%)	202 (28.9%)	
No	205 (29.3%)	148 (21.2%)	

In order to clearly distinguish factors that are significantly different, we have bolded p values less than 0.05.

**Table 2:** Logistic analysis of the association between TGIF2 expression and clinical characteristics.

Characteristics	Total (N)	Odds Ratio (95% CI)	P value
Age (<= 60 vs. > 60)	699	0.599 (0.412 - 0.871)	**0.007**
Gender (Male vs. Female)	699	1.104 (0.818 - 1.490)	0.519
WHO grade (G2 vs. G3&G4)	637	0.408 (0.292 - 0.571)	**< 0.001**
IDH status (WT vs. Mut)	689	4.159 (2.967 - 5.830)	**< 0.001**
1p/19q codeletion (Codel vs. Non-codel)	692	0.262 (0.179 - 0.384)	**< 0.001**
Primary therapy outcome (PD&SD vs. PR&CR)	465	1.381 (0.951 - 2.007)	0.090
Race (White vs. Asian&Black or African American)	686	1.333 (0.729 - 2.436)	0.350
Histological type (Glioblastoma vs. Astrocytoma&Oligoastrocytoma&Oligodendroglioma)	699	3.660 (2.498 - 5.365)	**< 0.001**

In order to clearly distinguish factors that are significantly different, we have bolded p values less than 0.05.

### 3.3 Prognostic value of TGIF2 expression in glioma

**Figure 3:**
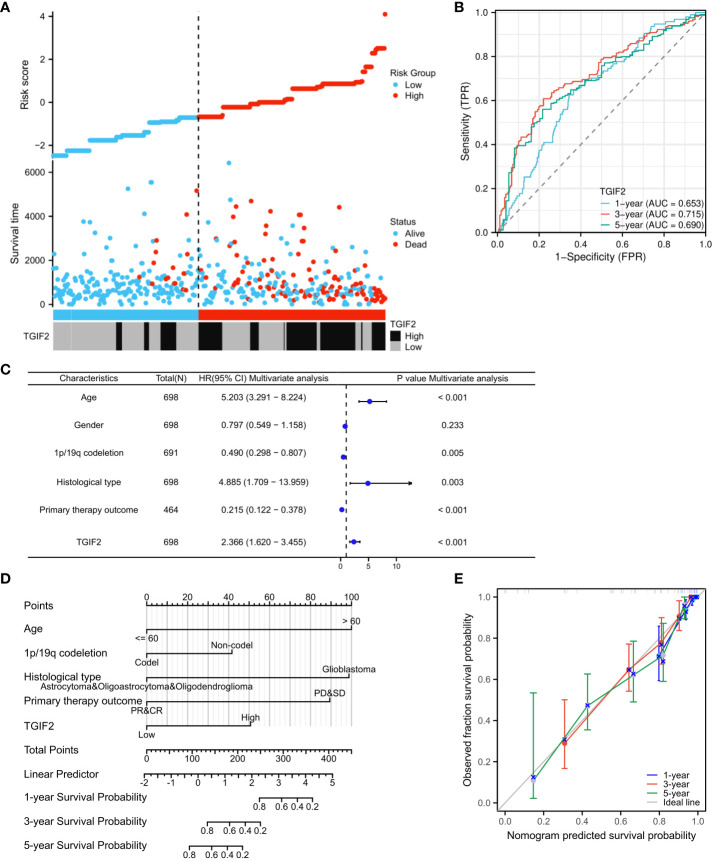
Prognostic value of TGIF2 expression level in glioma. **(A)** TGIF2 expression distribution and survival status. **(B)** Time-dependent ROC curves for TGIF2 expression in glioma. **(C)** Forest plot of OS by multivariate Cox regression analysis in glioma from TCGA database. **(D)** The nomogram for predicting 1-, 3-, or 5-year OS rates in patients with glioma. **(E)** The calibration curves for the nomogram.

We assessed the prognostic value of TGIF2 expression for glioma patients in the TCGA database. Firstly, analysis of TGIF2 expression distribution, survival status, and risk scores indicated that higher TGIF2 expression in the high-risk group correlated with increased mortality rates compared to the low-risk group (**Figure 3A**). Time-dependent ROC analysis demonstrated favorable predictive efficacy of TGIF2 expression for OS at 1-year (AUC = 0.653), 3-years (AUC = 0.715), and 5-years (AUC = 0.690) (**Figure 3B**). Univariate Cox regression analysis revealed an association between high TGIF2 expression and poorer OS (HR, 2.401; 95% confidence interval [CI], 1.867-3.089; p < 0.001) (**Table 3**). Multivariate Cox regression analysis, after confounding with other clinicopathologic variables, identified TGIF2 as an independent prognostic risk factor for glioma patients (HR, 2.366; 95% CI, 1.620-3.455; p < 0.001). Other clinicopathologic parameters, including age (HR, 5.203; 95% CI, 3.291-8.224; p < 0.001), 1p/19q codeletion (HR, 0.490; 95% CI, 0.298-0.807; p = 0.005), histological type (HR, 4.885; 95% CI, 1.709-13.959; p = 0.003), and primary therapy outcome (HR, 0.215; 95% CI, 1.620 -3.455; p < 0.001), also emerged as independent prognostic factors (**Table 3**; **Figure 3C**). A prognostic nomogram model, incorporating TGIF2 expression and other independent prognostic factors analyzed by Cox regression, was constructed for predicting OS at 1, 3, and 5 years (**Figure 3D**). Calibration curves assessed the predictive efficiency of the nomogram (**Figure 3E**).

**Table 3:** Univariate and multivariate cox regression analyses of clinical characteristics associated with overall survival.

Characteristics	Total(N)	Univariate analysis		Multivariate analysis	
		Hazard ratio (95% CI)	P value	Hazard ratio (95% CI)	P value
**Age**	698				
<= 60	555	Reference		Reference	
> 60	143	4.696 (3.620 - 6.093)	**< 0.001**	5.203 (3.291 - 8.224)	**< 0.001**
**Gender**	698				
Male	401	Reference		Reference	
Female	297	0.800 (0.627 - 1.021)	0.073	0.797 (0.549 - 1.158)	0.233
**Race**	685				
Asian&Black or African American	46	Reference			
White	639	0.817 (0.499 - 1.337)	0.421		
**1p/19q codeletion**	691				
Non-codel	520	Reference		Reference	
Codel	171	0.225 (0.147 - 0.346)	**< 0.001**	0.490 (0.298 - 0.807)	**0.005**
**Histological type**	698				
Astrocytoma& Oligoastrocytoma& Oligodendroglioma	530	Reference		Reference	
Glioblastoma	168	9.172 (7.052 - 11.929)	**< 0.001**	4.885 (1.709 - 13.959)	**0.003**
**Primary therapy outcome**	464				
PD&SD	260	Reference		Reference	
PR&CR	204	0.205 (0.117 - 0.359)	**< 0.001**	0.215 (0.122 - 0.378)	**< 0.001**
**TGIF2**	698				
Low	348	Reference		Reference	
High	350	2.401 (1.867 - 3.089)	**< 0.001**	2.366 (1.620 - 3.455)	**< 0.001**

In order to clearly distinguish factors that are significantly different, we have bolded p values less than 0.05.

**Figure 4:**
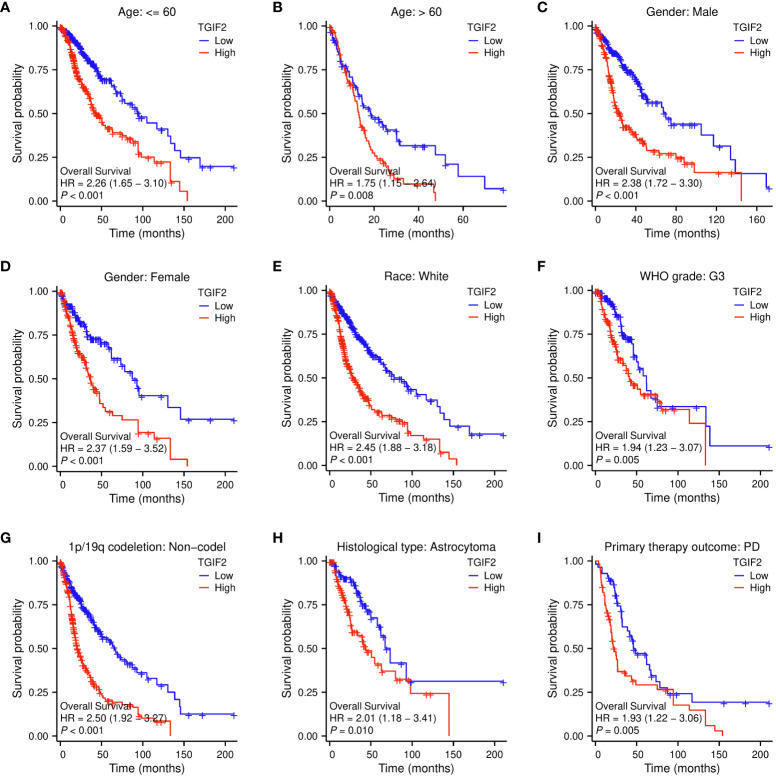
Correlations between TGIF2 expression level and OS in different clinicopathologic subgroups of glioma by Kaplan-Meier survival curve analysis. **(A)** Age ≤ 60. **(B)** Age > 60. **(C)** Gender: Male. **(D)** Gender: Female. **(E)** Race: White. **(F)** WHO grade: G3. **(G)** 1p/19q codeletion: non-codeletion. **(H)** Histological type: Astrocytoma. **(I)** Primary therapy outcome: PD.

Next, we explored the relationship between TGIF2 and prognosis (OS, DSS, and PFI) across different clinicopathologic subgroups of glioma. Kaplan-Meyer analysis revealed that higher TGIF2 expression associated with worse OS in subgroups including age >60, age ≤60, male, female, Race (White), WHO grade (G3), 1p/19q codeletion status (non-codel), histological type (astrocytoma), and primary therapy outcome (PD) (**Figures 4A–I**). For DSS, patients with higher TGIF2 expression had worse DSS in subgroups such as age >60, age ≤60, male, female, race (White), WHO grade (G3), 1p/19q codeletion status (non-codel), histological type (astrocytoma), primary therapy outcome (PD) and primary therapy outcome (SD) (**Supplementary Figures S1A–J**). For PFI, higher TGIF2 expression was associated with worse PFI in subgroups including age >60, age ≤60, male, female, race (White), WHO grade (G3), 1p/19q codeletion status (non-codel) (**Supplementary Figures S2A–G**).

### 3.4 Functional enrichment analysis of DEGs between TGIF2 high and low expression groups in glioma

**Figure 5:**
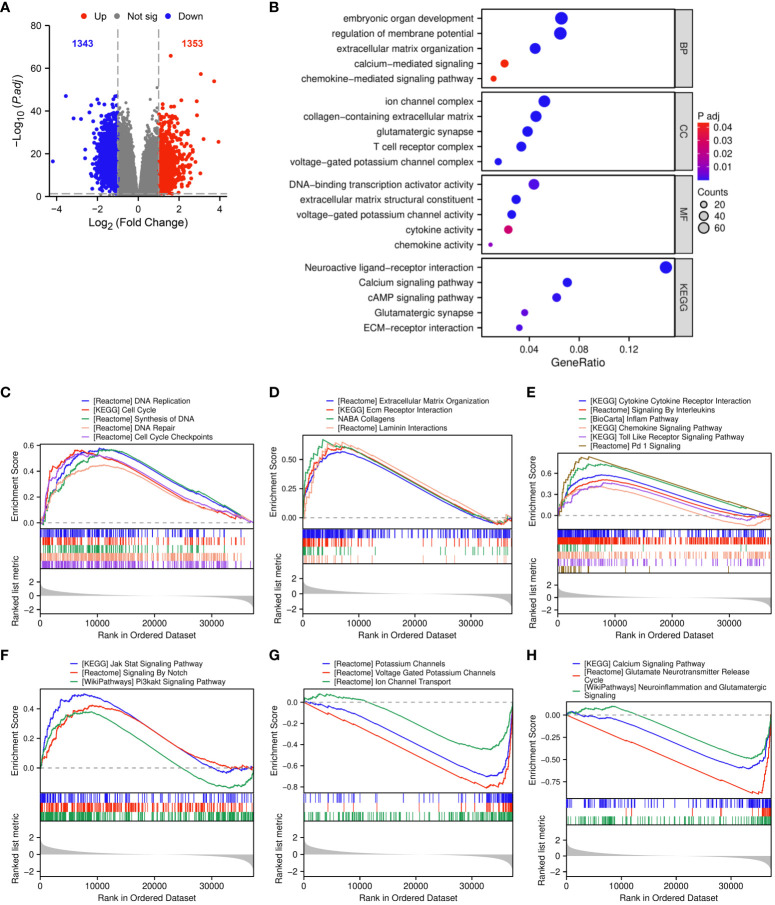
Functional enrichment analysis of DEGs between TGIF2 high and low expression groups. **(A)** The volcano plot of DEGs. Red represents upregulated, blue represents downregulated genes. **(B)** GO and KEGG pathway enrichment analysis of DEGs. **(C–H)** GSEA functional enrichment analysis.

We analyzed DEGs between TGIF2 high and low expression groups based on the median TGIF2 expression level. A total of 2696 DEGs were identified, comprising 1353 upregulated and 1343 downregulated genes (**Figure 5A**). In GO enrichment analysis, the biological process (BP) mainly contained embryonic organ development, regulation of membrane potential, extracellular matrix organization, calcium-mediated signaling and chemokine-mediated signaling pathway. The cellular component (CC) was enriched in ion channel complex, collagen-containing extracellular matrix, glutamatergic synapse, T cell receptor complex and voltage-gated potassium channel complex. The molecular function (MF) was mainly involved in DNA-binding transcription activator activity, extracellular matrix structural constituent, voltage-gated potassium channel activity, cytokine activity and chemokine activity. KEGG pathway enrichment analysis indicated that TGIF2 potential participation in the regulation of neuroactive ligand-receptor interaction, calcium signaling pathway, cAMP signaling pathway, glutamatergic synapse and ECM-receptor interaction (**Figure 5B**).

GSEA was applied to further investigate the biological functions of TGIF2. Upregulation of TGIF2 expression correlated with cell cycle and DNA replication pathways, including DNA replication, cell cycle, synthesis of DNA, DNA repair, and cell cycle checkpoints (**Figure 5C**). ECM-related functions, such as extracellular matrix organization, ECM receptor interaction, and collagens and laminin interactions, were enriched in the TGIF2 high-expression phenotype, suggesting involvement in ECM formation in glioma (**Figure 5D**). TGIF2 also impacted pathways related to tumor immunity, including cytokine cytokine receptor interaction, signaling by interleukins, inflam pathway, chemokine signaling pathway, toll-like receptor signaling pathway, and Pd-1 signaling (**Figure 5E**). Additionally, pathways related to tumor progression, such as Jak Stat signaling pathway, signaling by Notch, and Pi3kakt signaling pathway, were enriched (**Figure 5F**). Ion channel and glutamate signaling are facilitators of glioma cell invasion and migration (31, 32), yet they were negatively correlated with high TGIF2 expression (**Figures 5G, H**), which may be related to glioma grading (33, 34). These findings suggest that TGIF2 may play a role in glioma cell proliferation, invasion and migration, and tumor immunity, making it a promising target for glioma treatment.

### 3.5 Correlation between TGIF2 expression and immune cell infiltration in glioma

**Figure 6:**
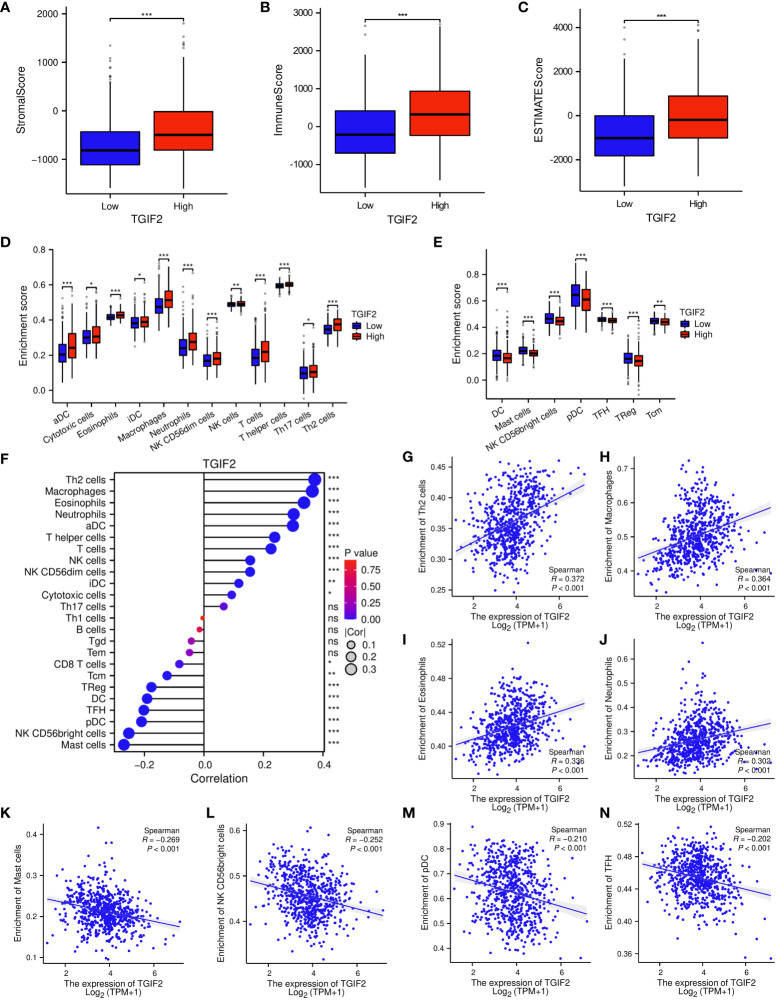
Correlations between TGIF2 expression and immune cell infiltration in glioma. **(A–C)** Comparison of StromalScore, ImmuneScore, and EstimateScore between TGIF2 high and low expression groups. **(D, E)** Comparison of immune cell enrichment scores in high and low TGIF2 expression groups. **(F)** The lollipop chart showing the correlations between the relative abundances of 24 immune cells and TGIF2 expression levels. **(G–N)** Scatterplots demonstrating the positive correlation of TGIF2 expression with Th2 cells, macrophages, eosinophils, and neutrophils, and the negative correlation with mast cells, NK CD56bright cells, pDC cells, and TFH cells. ∗p < 0.05, ∗∗p < 0.01, ∗∗∗p < 0.001, ns, not significant.

To unravel the intricate relationship between TGIF2 expression and the tumor immune response in glioma, we meticulously analyzed the disparities in the glioma immune microenvironment between high and low TGIF2 expression groups. The outcomes unveiled a significant elevation in stromal scores (**Figure 6A**), immunity scores (**Figure 6B**), and estimated scores (**Figure 6C**) in glioma patients with high TGIF2 expression. Furthermore, multiple immune cell subtypes were significantly enriched in the TGIF2 high-expression group, including aDC, cytotoxic cells, eosinophils, iDC, macrophages, neutrophils, NK CD56dim cells, NK cells, T cells, T helper cells, Th17 cells, and Th2 cells (**Figure 6D**). In contrast, the TGIF2 low-expression group exhibited higher enrichment of DC, mast cells, NK CD56bright cells, pDC, TFH, TReg, and Tcm (**Figure 6E**). Subsequently, we further delved into the association between TGIF2 expression levels and the infiltration levels of different immune cells in gliomas. The results demonstrated a positive correlation between TGIF2 expression and the infiltration levels of Th2 cells, macrophages, eosinophils and neutrophils, *etc.* Nevertheless, there was a negative correlation with infiltration levels of mast cells, NK CD56bright cells, pDC and TFH, *etc.* (**Figures 6F–N**). In addition, we investigated the correlation between TGIF2 and various immunoregulatory molecules to further understand its immune modulating function. These immunoregulatory genes include antigen presentation, cell adhesion, co-inhibitory, co-stimulatory, ligand, receptor, and other according to the classification of Thorsson et al. (23). The findings indicated a positive correlation between TGIF2 expression and most immunoregulatory genes, implying a potential role for TGIF2 in regulating glioma immune infiltration (**Supplementary Figure S3**).

### 3.6 Analysis of genes coexpressed with TGIF2 in glioma

**Figure 7:**
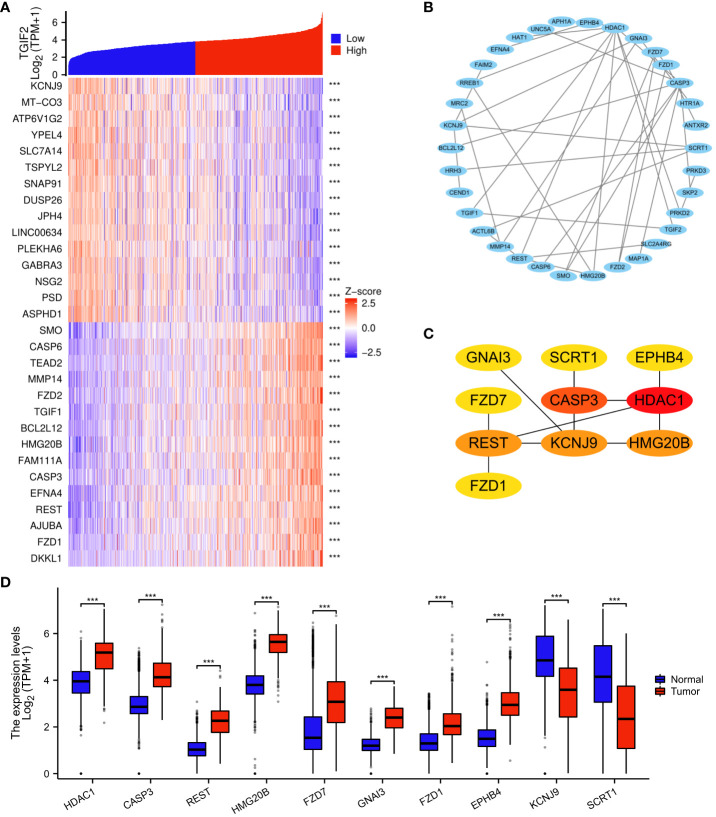
Analysis of genes coexpressed with TGIF2 in glioma. **(A)** Heatmap showing the 30 genes in glioma that were 15 positively and 15 negatively related to TGIF2. **(B)** PPI network of top 50 protein-coding genes positively and negatively correlated with TGIF2. **(C)** The top 10 hub genes of the PPI network in **(B)**. **(D)** The expression levels of the top 10 hub genes in normal and glioma tissues in TCGA and GTEx database. ∗∗∗p < 0.001.

**Figure 8:**
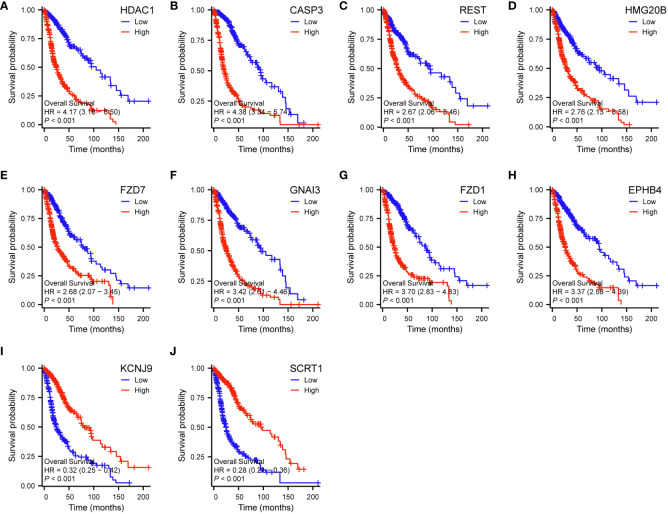
Correlations between the top 10 hub genes and OS of glioma patients in TCGA database by Kaplan-Meier survival curve analysis. **(A)** HDAC1. **(B)** CASP3. **(C)** REST. **(D)** HMG20B. **(E)** FZD7. **(F)** GNAI3. **(G)** FZD1. **(H)** EPHB4. **(I)** KCNJ9. **(J)** SCRT1.

**Figure 9:**
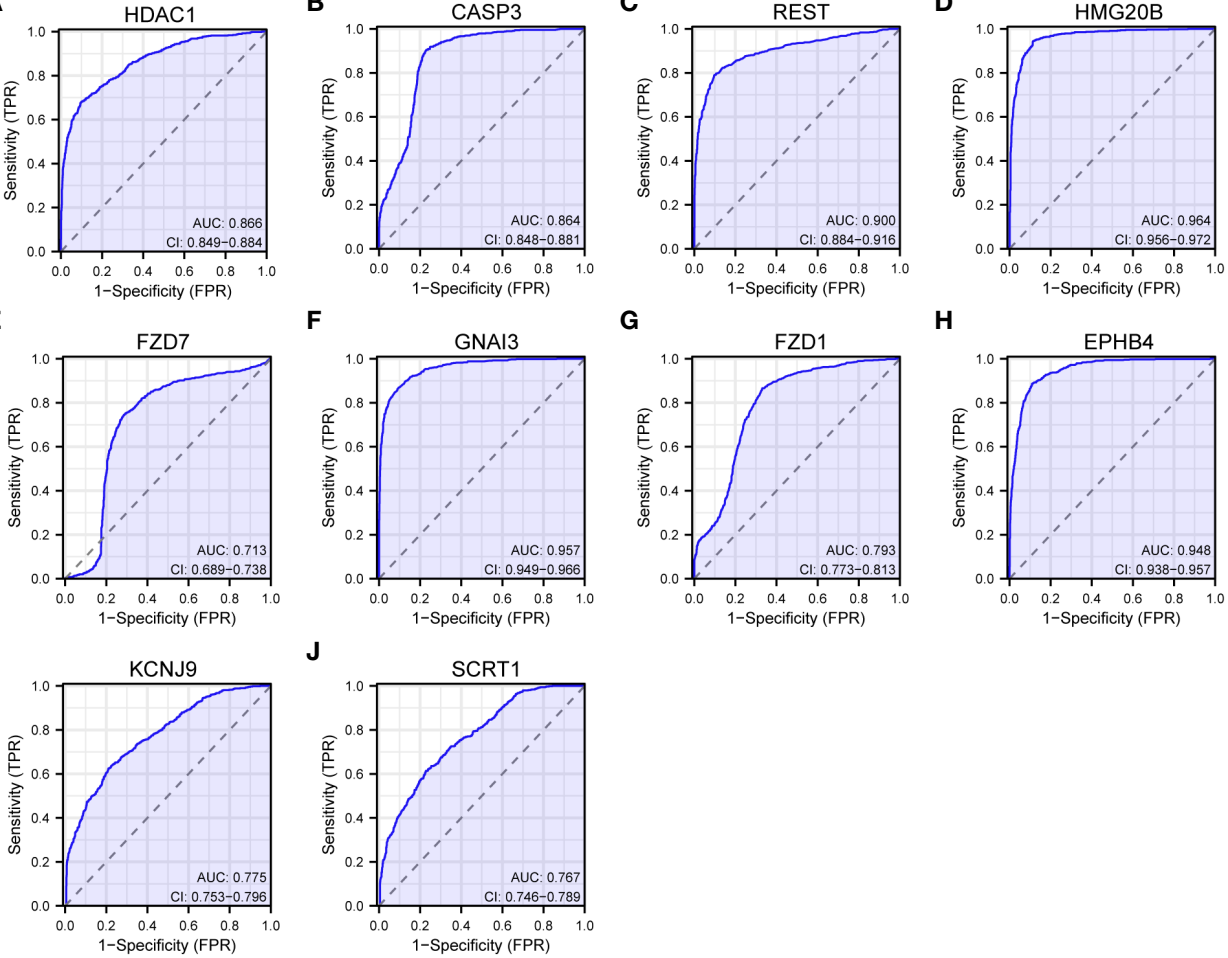
ROC curves of the top 10 hub genes. **(A)** HDAC1. **(B)** CASP3. **(C)** REST. **(D)** HMG20B. **(E)** FZD7. **(F)** GNAI3. **(G)** FZD1. **(H)** EPHB4. **(I)** KCNJ9. **(J)** SCRT1.

The analysis of genes coexpressed with TGIF2 in glioma provided valuable insights into potential regulatory networks and prognostic markers. The coexpression heatmap demonstrated the top 15 genes that were positively and negatively correlated with TGIF2 (**Figure 7A**). We performed PPI network analysis of the top 50 protein-coding genes positively and negatively correlated with TGIF2 using the STRING database and Cytoscape (**Figure 7B**) and identified the top 10 hub genes using the CytoHubba plugin (HDAC1, CASP3, REST, HMG20B, FZD7, GNAI3, FZD1, EPHB4, KCNJ9, SCRT1) (**Table 4**, Figure 7C). The top 10 hub genes highlighted potential central regulators in the context of TGIF2-associated glioma biology. Among these 10 genes HDAC1, CASP3, REST, HMG20B, FZD7,GNAI3, FZD1 and EPHB4 had elevated expression in tumor group compared to the normal group, while KCNJ9 and SCRT1 were decreased (**Figure 7D**). Kaplan-Meyer analysis showed that high expression of HDAC1, CASP3, REST, HMG20B, FZD7,GNAI3, FZD1 and EPHB4 was associated with worse OS, DSS and PFI, whereas high expression of KCNJ9 and SCRT1 suggested better OS, DSS and PFI (**Figures 8A–J**; **Supplementary Figures S3A–J**, **S4A–J**). ROC analysis further validated the good diagnostic capabilities of these 10 hub genes in glioma (**Figures 9A–J**). In summary, this comprehensive analysis not only revealed potential regulatory networks with TGIF2 in glioma but also highlighted a promising gene cluster for prognostic assessment. Integrating these hub genes with TGIF2 may enhance the accuracy of prognostic predictions in glioma patients.

**Table 4:** The top 10 hub genes identified in the PPI network.

Gene symbol	Gene description
HDAC1	Histone deacetylase 1
CASP3	Caspase 3
REST	RE1 silencing transcription factor
HMG20B	High mobility group 20B
FZD7	Frizzled class receptor 7
GNAI3	G protein subunit alpha I3
FZD1	Frizzled class receptor 1
EPHB4	EPH receptor B4
KCNJ9	Potassium inwardly rectifying channel subfamily J member 9
SCRT1	Scratch family transcriptional repressor 1

### 3.7 Knockdown and knockout of TGIF2 inhibits glioma cell invasion,migration and EMT *in vitro*

**Figure 10:**
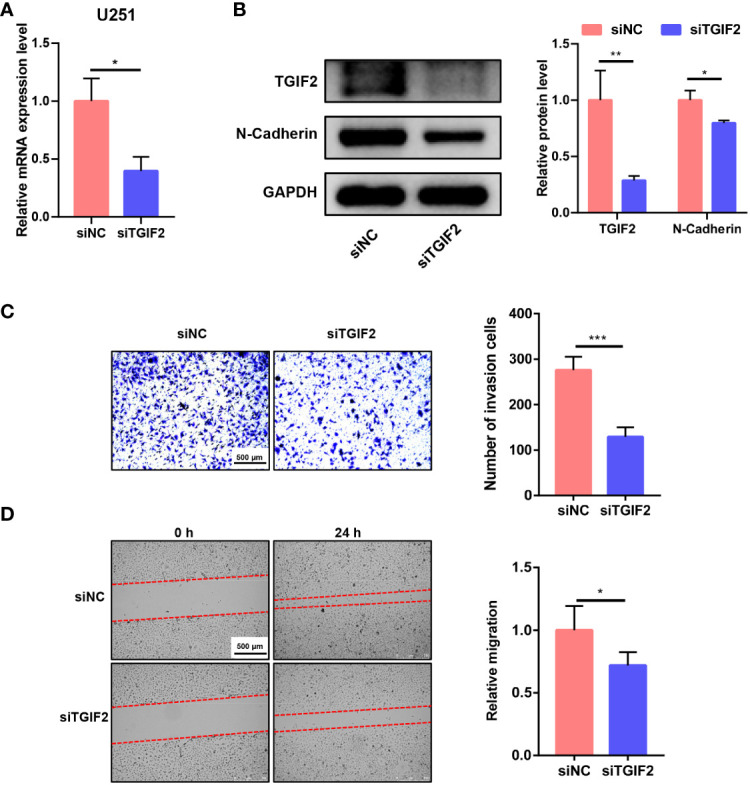
Knockdown of TGIF2 inhibits glioma cell invasion, migration and EMT *in vitro*. **(A)** Bar graph demonstrating the efficiency of siRNA knockdown of TGIF2 mRNA in U251 cells by RT-qPCR. **(B)** Western blot assay showing that siRNA effectively knocked down TGIF2 protein, and siTGIF2 downregulated N-cadherin protein expression in U251 cells. **(C)** Transwell assay demonstrating changes in the number of cells invaded after knockdown of TGIF2. Scale bar, 500 μm. **(D)** Scratch wound-healing assays were utilized to compare the distance of cell migration between TGIF2-inhibited group and control group at 0h and 24h after scratching. Scale bar, 500 μm. ∗p < 0.05, ∗∗p < 0.01, ∗∗∗p < 0.001.

**Figure 11:**
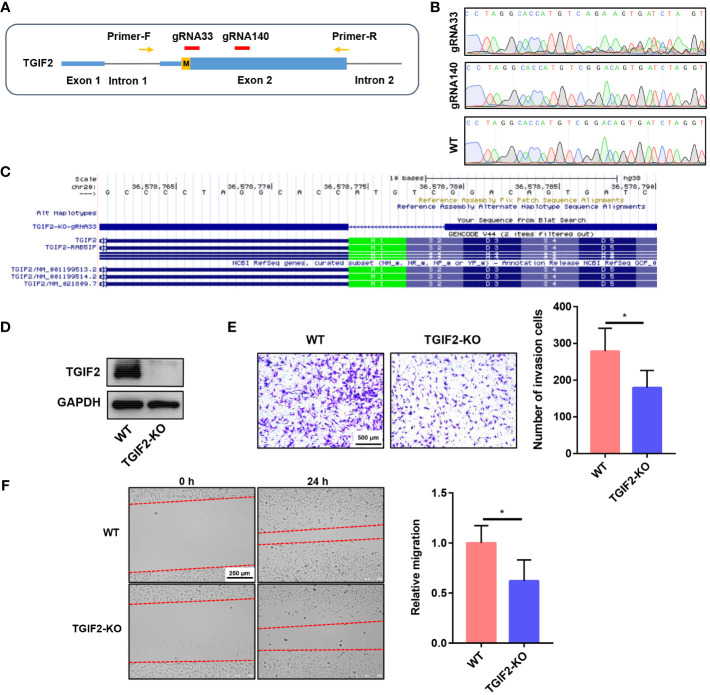
Knockout of TGIF2 suppresses glioma cell invasion, migration and EMT. **(A)** The gRNA sequences designed targeting exon 2 of TGIF2. **(B)** The editing efficiency of gRNA33 and gRNA140 on TGIF2 was verified in 293T cells utilizing Sanger sequencing. **(C)** TGIF2-gRNA33 in the UCSC browser. **(D)** Western blot assay showing the knockout efficiency of TGIF2 protein. WT, Wild type; KO, Knockout. **(E)** Transwell assays showing changes in the number of cells invaded after knockout of TGIF2. Scale bar, 500 μm. **(F)** Scratch wound-healing assays were utilized to compare the distance of cell migration between TGIF2-knockout group and control group at 0h and 24h after scratching. Scale bar, 250 μm. ∗p < 0.05.

We explored the function of TGIF2 *in vitro*. Initially, we employed siRNA to knock down TGIF2 expression in the U251 glioma cell line. Both RT-qPCR and Western blot analyses confirmed the efficacy of TGIF2 knockdown, ensuring the reliability of subsequent experiments (**Figures 10A, B**). Subsequent transwell assays and scratch wound-healing assays revealed that TGIF2 inhibition suppressed the invasion and migration of U251 cells (**Figures 10C, D**). Furthermore, our investigations revealed a downregulation of N-Cadherin, a well-established marker of EMT, in the TGIF2-inhibited group (**Figure 10B**). This observation suggests that TGIF2 may play a role in promoting the EMT phenotype, thereby influencing glioma cell invasion and migration through the regulation of N-Cadherin expression. We used CRISPR/Cas9 gene editing to further validate the function of TGIF2. We designed gRNA sequences gRNA33 and gRNA140 targeting exon 2 of TGIF2, cloned them into the CRISPER Cas9 plasmid PX459 and verified the editing efficiency on TGIF2 (**Figure 11A**). Next, we transfected the PX459-gRNA33 plasmid, which was effective in editing TGIF2, into glioma U251 cells, and screened for TGIF2 knockout cell lines using Sanger sequencing (**Figures 11B, C**). Western blot analyses verified the knockout efficiency for TGIF2 of the PX459-gRNA33 plasmid in U251 cells (**Figure 11D**). Subsequently, transwell assays and scratch wound-healing assays showed that knockout of TGIF2 inhibited invasion and migration of U251 cells (**Figures 11E, F**). In addition, the expression of the signature genes of EMT (CDH2, TWIST1 and TWIST2) were also suppressed in the TGIF2-knockout group (**Supplementary Figure S6**). These results strengthen the evidence that TGIF2 promotes glioma cell invasion, migration and EMT.

## 4 Discussion

Glioma, recognized as the most lethal adult intracranial primary tumor with low overall survival rates (1), continues to pose formidable challenges despite advancements in treatment modalities (35). There is an imperative need for novel early diagnostic and prognostic targets to address the pressing need for glioma treatment. Numerous studies have been devoted to exploring biomarkers in order to open new avenues for glioma treatment (28–30, 36, 37). The 1p/19q codeletion and IDH mutation emerged as the initial biomarkers, widely employed in clinical glioma diagnosis and have been associated with a variety of prognostic factors to establish effective clinical prediction model (38–40). In this study, we focus on the transcription factor TGIF2 and propose for the first time its potential as a promising diagnostic and prognostic target for glioma. Our investigation demonstrated that TGIF2 expression significantly surpasses normal tissue levels in gliomas and correlates with adverse prognosis and malignant phenotypes. We established high TGIF2 expression as an independent risk factor for OS in glioma patients, as validated by multivariate Cox regression analysis. Moreover, we constructed a prognostic nomogram model of TGIF2 versus age, 1p/19q codeletion, histological type, and primary therapy outcome, enhancing the accuracy of predicting glioma risk factors for future clinical applications. Survival analysis across various clinicopathologic subgroups consistently demonstrated the significant association of TGIF2 with poor OS, DSS and PFI, underscoring its effective prognostic value. These findings collectively advocate TGIF2 as a promising biomarker for both diagnosing and predicting outcomes in glioma patients, offering potential avenues for improved therapeutic strategies and patient care.

To further elucidate the biological function of TGIF2 in glioma development, we divided glioma patients into high and low expression groups based on the median TGIF2 expression, analyzed the differentially expressed genes in the two groups, and performed functional enrichment analysis. Cell hyperproliferation is a common characteristic of the vast majority of tumors, and consistently, we observed multiple items related to cell cycle and DNA synthesis and repair associated with high TGIF2 expression. In addition, our attention was drawn to several ECM-related items. Elevated expression of ECM components creates an impermeable microenvironment for glioma to evade drug and immune attacks, and contributes to tumor cell migration and invasion (41). The positive correlation between the high expression of ECM and TGIF2 suggests that TGIF2 may be involved in the formation of the ECM and thus promote the migration and invasion of glioma cells. In addition, signaling pathways closely related to tumorigenesis, such as Jak Stat signaling pathway (42), Notch signaling pathway (43), and Pi3k akt signaling pathway (44) were associated with increased expression of TGIF2. Notably, NOTCH signaling was reported to be associated with EMT, and the promotion of EMT in glioma cell by TGIF2 has been reported in glioma (21, 45), speculating a potential regulatory crosstalk between TGIF2 and NOTCH signaling. These findings suggest that TGIF2 may play a multifaceted role in glioma progression, influencing processes such as cell cycle regulation, ECM formation, and engagement with key signaling pathways. These insights contribute to a deeper understanding of TGIF2’s impact on glioma development and progression.

The tumor immune microenvironment plays a crucial role in glioma progression and therapy response (46). Immune infiltration is intricately linked to the immune escape of tumor cells and exerts regulatory control over the remodeling of the tumor microenvironment. Immunotherapy, which removes tumor cells by altering or modulating the autoimmune system, has emerged as a powerful anticancer strategy compared to conventional therapies such as surgery, radiotherapy and chemotherapy. Several immunotherapeutic approaches have shown promise in glioma, including immune checkpoint inhibitors (ICIs), cancer vaccines, adoptive cell transfer (ACT), CAR-T cell therapy, and more (47). Additionally, the integration of nanomaterials, such as dendrimers, multifunctional nano-adjuvants, and nanoprobes, has expanded the possibilities in cancer immunotherapy (48–50). Approaches like near-infrared photoimmunotherapy have even progressed to the clinical study stage (51). Despite these advancements, each immunotherapeutic strategy has its limitations, and there is a continued need to explore and understand the intricate regulation of the glioma immune microenvironment. In this study, functional enrichment analysis showed that high TGIF2 expression was positively correlated with immune-related pathways such as chemokines, cytokines, interleukins, inflammation, toll like receptor and Pd 1. Notably, PD-L1 expression in glioma has been associated with WHO classification, positioning it as a potential biomarker (52, 53). The enrichment of the Pd-1 signaling pathway suggested a potential involvement of TGIF2 in the regulation of the PD-1/PD-L1 axis. This signaling axis is a critical component of immune checkpoint regulation and can influence the immune response against tumors. To further explore the relationship between TGIF2 and tumor immunity, we analyzed the infiltration level of immune cells in the microenvironment of glioma tumors. Our results showed that TGIF2 was positively correlated with Th2, macrophages, eosinophils and neutrophils, *etc.*, while displaying a negative correlation with mast cells, NK CD56bright cells, pDC, and TFH, *etc.* The balance of Th1/Th2 is an important mechanism leading to immune evasion of tumors (54, 55), therefore, TGIF2-mediated Th2 enrichment may be one of the potential factors for tumor cells to evade immune surveillance. On the contrary, TFH cells were associated with anti-tumor immunity (56), and the negative correlation observed between TGIF2 and TFH cells suggests a potential contribution of TGIF2 to tumor immune escape. The implications of these findings underscore the potential importance of TGIF2 in shaping the glioma immune microenvironment. However, the precise role and underlying mechanisms necessitate further elucidation through dedicated biological experiments.

We constructed a PPI network of the top 50 genes positively and negatively correlated with TGIF2 respectively and identified 10 hub genes (HDAC1, CASP3, REST, HMG20B, FZD7, GNAI3, FZD1, EPHB4, KCNJ9, SCRT1) which have good diagnostic ability in glioma. Among them, high expression of HDAC1, CASP3, REST, HMG20B, FZD7, GNAI3, FZD1 and EPHB4 were associated with worse OS, DSS and PFI, while high expression of KCNJ9 and SCRT1 suggested better OS, DSS and PFI. Consistent with our results, high expression of HDAC1, CASP3, REST, GNAI3, FZD1, EPHB4 was found to correlate with poor prognosis of glioma, and inhibitors of HDAC1 inhibited the EMT process in glioma cells thereby affecting cell migration and invasion (57–62). FZD7, linked to glioma cell motility and invasiveness, aligns with our observation of TGIF2 promoting glioma cell migration (63). Collectively, these results suggest a potential co-regulation of migration and invasion in glioma by TGIF2 and these identified hub genes.

Although this study demonstrated an association between TGIF2 and glioma, there are still some limitations and shortcomings of this study. Since we utilized transcriptome sequencing data included in public databases and patients’ clinicopathologic information for bioinformatics analysis, there were certain biases caused by confounding factors, and future prospective studies will be helpful in this regard. Meanwhile, our study needs more clinical samples to validate the aberrant expression of TGIF2 to improve reliability. In addition, combined with previous studies, our *in vitro* experiments have provided a preliminary exploration of the role of TGIF2 in glioma migration, invasion and EMT, and the specific mechanisms and more functions in glioma need to be further investigated.

In conclusion, our results suggest that TGIF2 can be used as a promising new indicator for predicting the malignant phenotypes and clinical prognosis of glioma patients, and correlates with immune infiltration and EMT phenotype.

## Data availability statement

The datasets presented in this study can be found in online repositories. The names of the repository/repositories and accession number(s) can be found in the article/**Supplementary Material**.

## Ethics statement

Ethical approval was not required for the studies on humans in accordance with the local legislation and institutional requirements because only commercially available established cell lines were used.

## Author contributions

WZ: Writing – original draft, Conceptualization, Data curation, Investigation, Methodology, Project administration, Software, Visualization, Writing – review & editing. LZ: Writing – review & editing, Data curation, Supervision, Funding acquisition. HD: Writing – review & editing, Investigation, Methodology, Validation. HP: Conceptualization, Supervision, Validation, Visualization, Writing – original draft, Writing – review & editing, Project administration.
