# Supplementary material for: TGIF2 is a potential biomarker for diagnosis and prognosis of glioma
Source: Front Immunol. 2024 Feb 26;15:1356833. doi: 10.3389/fimmu.2024.1356833 (PMC11020094; doi:10.3389/fimmu.2024.1356833)
Supplement: Supplementary file 1 [file DataSheet_1.docx]

Supplementary Material

TGIF2 is a potential biomarker for diagnosis and prognosis of glioma

Wan Zhang, Long Zhang, Huanhuan Dong, Hang Peng

*** Correspondence:** Hang Peng: [hangpeng21@126.com](mailto:hangpeng21@126.com);

*** Co-correspondence:** Long Zhang: [longzhang@xjtu.edu.cn](mailto:longzhang@xjtu.edu.cn)

# Supplementary Figures and Tables

## Supplementary Figures

**
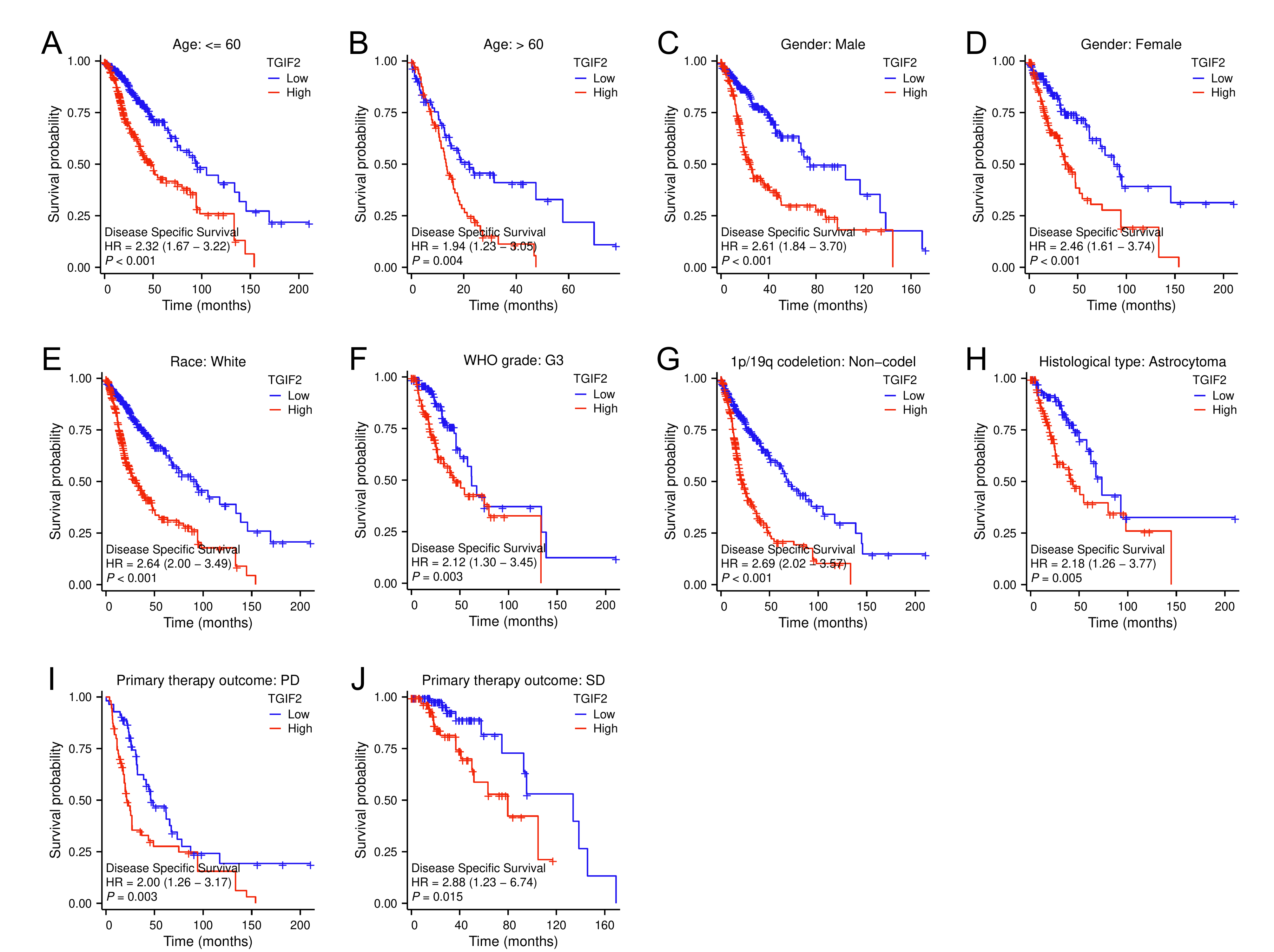
Supplementary Figure 1.** Correlations between TGIF2 expression level and DSS in different clinicopathologic subgroups of glioma by Kaplan-Meier survival curve analysis. **(A)** Age ≤ 60. **(B)** Age > 60. **(C)** Gender: Male. **(D)** Gender: Female. **(E)** Race: White. **(F)** WHO grade: G3. **(G)** 1p/19q codeletion: non-codeletion. **(H)** Histological type: Astrocytoma. **(I)** Primary therapy outcome: PD. **(J)** Primary therapy outcome: SD.

**
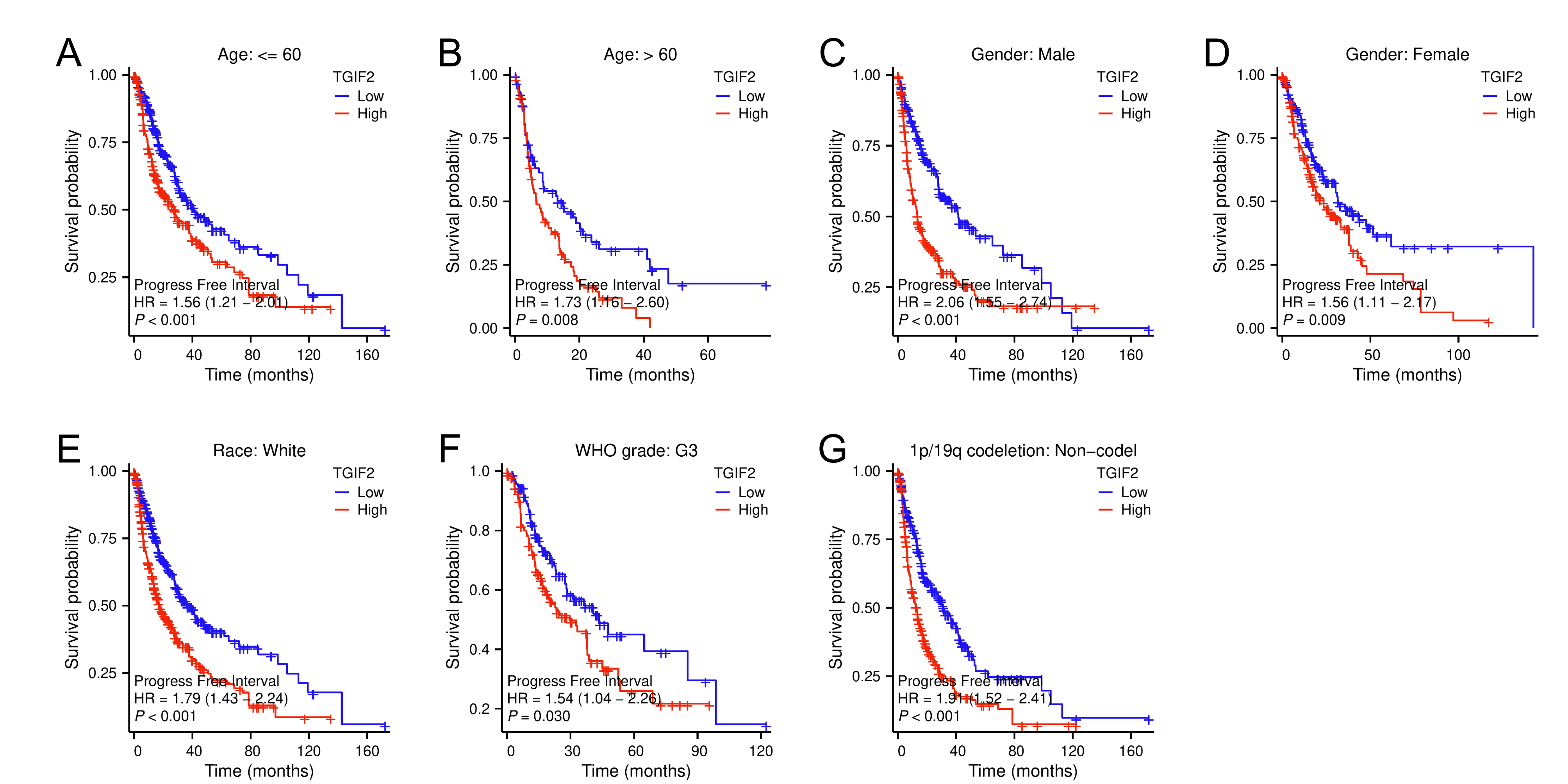
Supplementary Figure 2.** Correlations between TGIF2 expression level and DSS in different clinicopathologic subgroups of glioma by Kaplan-Meier survival curve analysis. **(A)** Age ≤ 60. **(B)** Age > 60. **(C)** Gender: Male. **(D)** Gender: Female. **(E)** Race: White. **(F)** WHO grade: G3. **(G)** 1p/19q codeletion: non-codeletion.


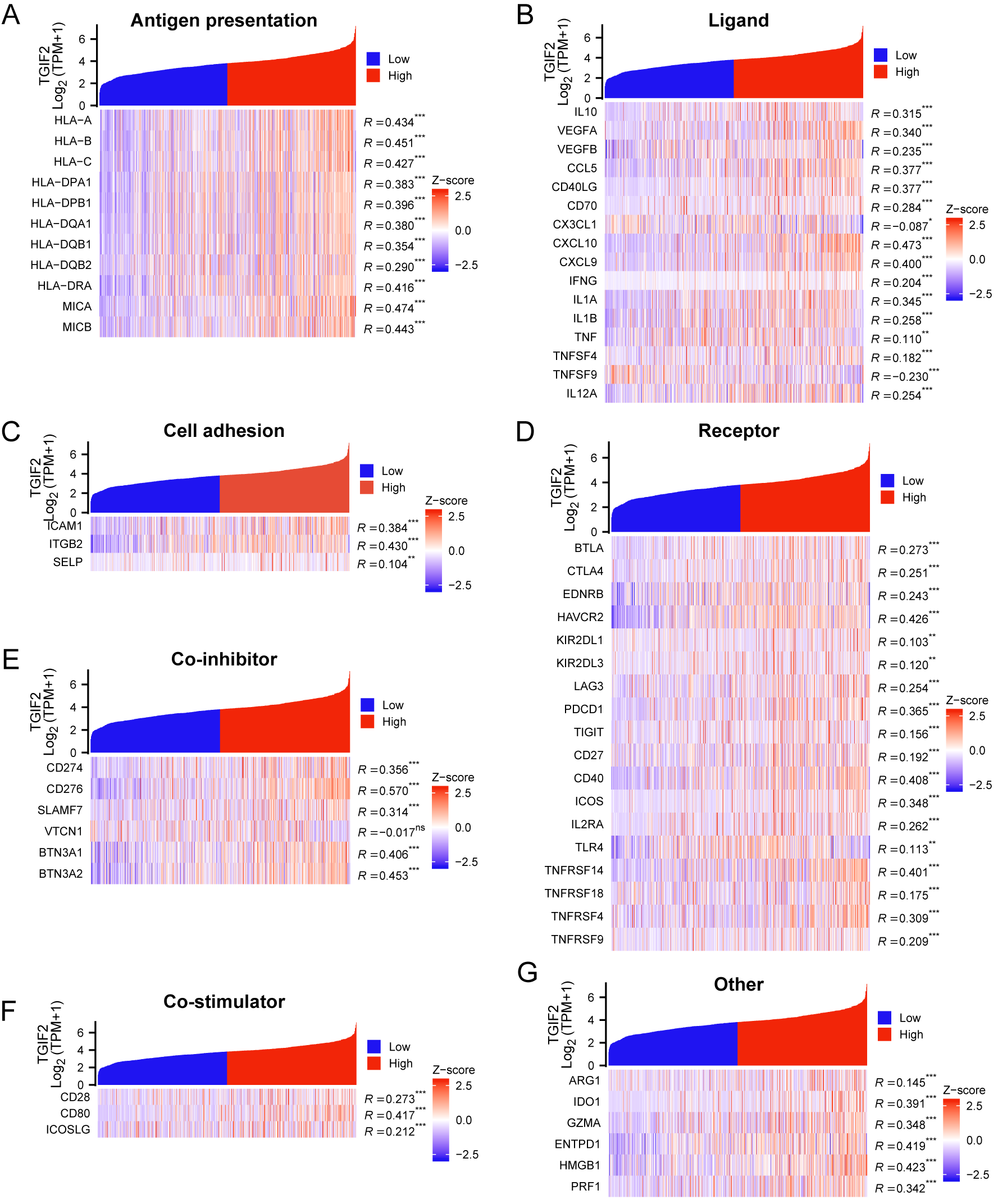


**Supplementary Figure 3.** Analyses of correlations between TGIF2 and immunoregulatory genes in glioma in TCGA database **(A-G)**. ∗p < 0.05, ∗∗p < 0.01, ∗∗∗p < 0.001, ns, not significant.

**
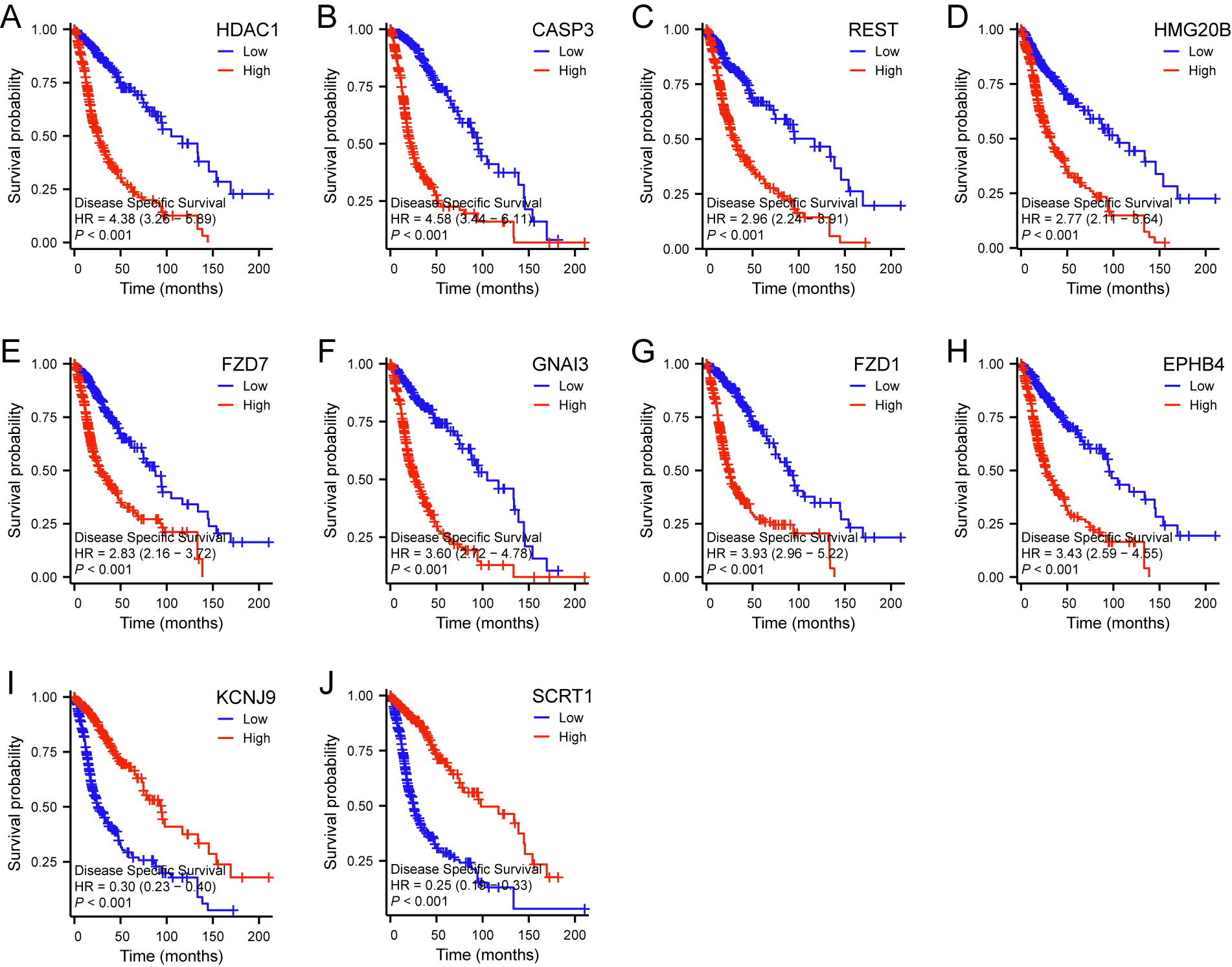
**

**Supplementary Figure 4.** Correlations between the top 10 hub genes and DSS of glioma patients in TCGA database by Kaplan-Meier survival curve analysis. **(A)** HDAC1. **(B)** CASP3. **(C)** REST. **(D)** HMG20B. **(E)** FZD7. **(F)** GNAI3. **(G)** FZD1. **(H)** EPHB4. **(I)** KCNJ9. **(J)** SCRT1.


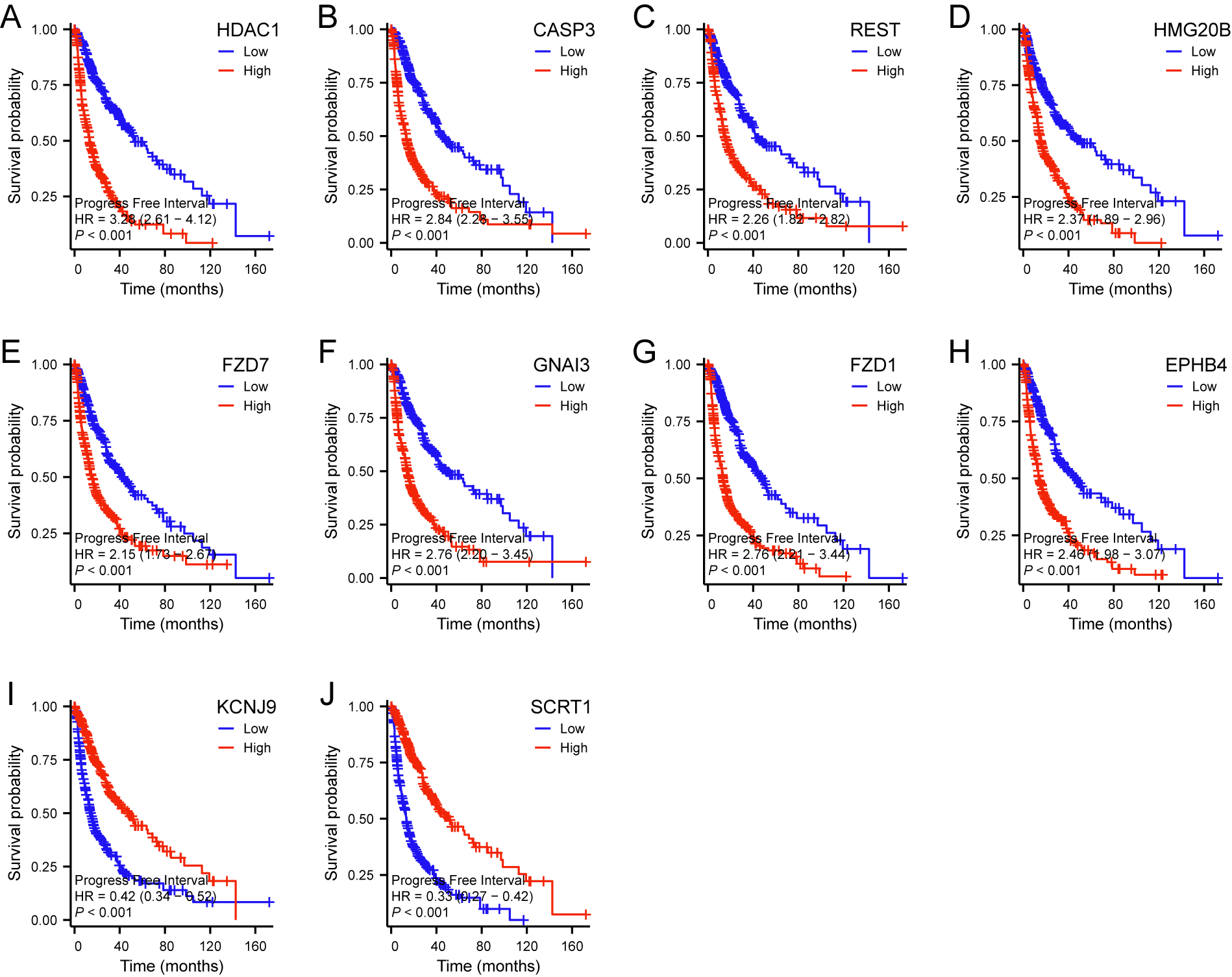


**Supplementary Figure 5.** Correlations between the top 10 hub genes and PFI of glioma patients in TCGA database by Kaplan-Meier survival curve analysis. **(A)** HDAC1. **(B)** CASP3. **(C)** REST. **(D)** HMG20B. **(E)** FZD7. **(F)** GNAI3. **(G)** FZD1. **(H)** EPHB4. **(I)** KCNJ9. **(J)** SCRT1.


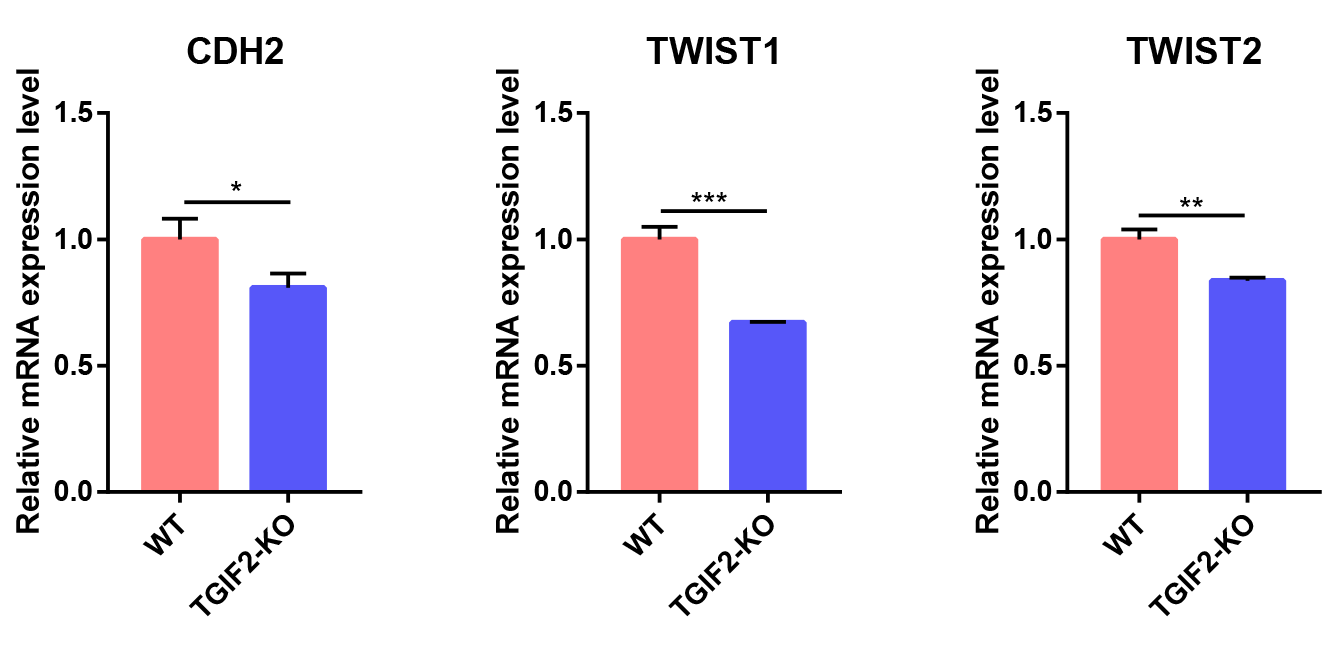


**Supplementary Figure 6.** Bar graphs demonstrating that EMT signature genes (CDH2, TWIST1 and TWIST2) were downregulated in the TGIF2-knockout group compared to the control group. ∗p < 0.05, ∗∗p < 0.01, ∗∗∗p < 0.001.
